# Efficacy of cognitive rehabilitation training combined with multisensory stimulation on cognitive function and quality of life in patients after intracerebral hemorrhage surgery

**DOI:** 10.12669/pjms.42.3.12775

**Published:** 2026-03

**Authors:** Yuanyuan Li, Xiaofei Li, Bonan Zhao

**Affiliations:** 1 Yuanyuan Li, Neurosurgery Cerebrovascular Disease, Spinal Cord Ward, Baoding No.1 Central Hospital, Baoding 071000, Hebei, China; 2 Xiaofei Li, Neurosurgery Cerebrovascular Disease, Spinal Cord Ward, Baoding No.1 Central Hospital, Baoding 071000, Hebei, China; 3 Bonan Zhao, Neurosurgery Cerebrovascular Disease, Spinal Cord Ward, Baoding No.1 Central Hospital, Baoding 071000, Hebei, China

**Keywords:** Cognitive function, Cognitive rehabilitation training, Multisensory stimulation, Post-cerebral hemorrhage surgery, Quality of life

## Abstract

**Objective:**

To evaluate the efficacy of cognitive rehabilitation training(CRT) combined with multisensory stimulation(MSS), aiming to provide an effective intervention strategy and theoretical basis for improving postoperative rehabilitation outcomes in this patient population.

**Methodology:**

This was a retrospective study. A total of 80 patients who underwent surgical treatment for Intracranial Hemorrhage (ICH) at the Department of Neurosurgery, Baoding No.1 Central Hospital between January to April 2025 were enrolled and randomly assigned to either the observation group or the control group (*n=* 40 each). All patients received standard postoperative care. In addition to routine rehabilitation guidance provided to the control group, the observation group received a combined regimen of CRT and MSS. Key outcome measures were assessed at one and three months postoperative.

**Results:**

At both one and three months postoperative, patients in the observation group showed significantly higher scores on the Montreal Cognitive Assessment compared with those in the control group(*P<* 0.05). At three months postoperative, quality of life, as measured by the SF-36, was significantly better in the observation group(*P<* 0.05). Additionally, scores on the Self-Rating Anxiety Scale and Self-Rating Depression Scale were significantly lower in the observation group at both time points(*P<* 0.05, respectively). The degree of neurological impairment, as measured by the National Institutes of Health Stroke Scale, was also significantly reduced in the observation group compared with the control group at both one and three months postoperative(*P<* 0.05, respectively).

**Conclusion:**

The combination of CRT and MSS is an effective therapeutic approach for enhancing cognitive function, improving quality of life and alleviating psychological distress in patients after ICH surgery.

## INTRODUCTION

Intracerebral hemorrhage (ICH) is a common and severe neurological condition characterized by high incidence, high disability and high mortality rates.^1^,^2^ Although advancements in medical and surgical techniques have led to a gradual decline in ICH-related mortality, postoperative cognitive impairment and diminished quality of life remain prevalent and distressing issues for many patients and their families.^3^ Cognitive impairment not only compromises patients’ ability to perform activities of daily living (ADL) but also significantly impedes psychological well-being and social reintegration.^4^ Therefore, identifying effective rehabilitation approaches to improve cognitive outcomes and quality of life in patients after ICH surgery holds substantial clinical and societal importance. Conventional rehabilitation programs typically focus on motor function recovery, often neglecting cognitive rehabilitation.^5^

In recent years, with growing insights into neural plasticity, cognitive rehabilitation training (CRT) and multisensory stimulation (MSS) have emerged as two promising approaches in the rehabilitation of neurological disorders.^6^,^7^ However, existing research on the combined application of CRT and MSS in patients after ICH surgery remains limited and lacks systematic exploration.^8^ This study investigated the effects of CRT plus MSS on cognitive function, quality of life, mental state and neurological impairment in patients after ICH surgery, aiming to provide a theoretically grounded and clinically effective strategy to enhance rehabilitation outcomes. Ultimately, this study aspires to offer new insights and practical approaches for postoperative rehabilitation, supporting more comprehensive recovery and improved quality of life in patients with ICH.

## METHODOLOGY

This was a retrospective study. A total of 80 patients who underwent surgical treatment for ICH in the Department of Neurosurgery between January and April 2025 were enrolled in this study. Patients were randomly assigned to either the observation group or the control group, with 40 cases in each group.

### Ethical approval:

The study was approved by the Institutional Ethics Committee of Baoding No.1 Central Hospital (No.:[2025]055; date: April 18, 2025) and written informed consent was obtained from all participants.

### Diagnostic criteria:

Diagnosis of ICH was established based on the Chinese Guidelines for Diagnosis and Treatment of Intracerebral Hemorrhage(2019):

- Acute onset with symptoms typically peaking within minutes to hours.
- Presence of symptoms such as headache, vomiting, varying degrees of consciousness disturbance and limb paralysis.
- Neuroimaging findings showing high-density lesions within the brain parenchyma on computed tomography scans or abnormal magnetic resonance imaging signals.
- Inclusion criteria:
- Age between 18 and 75 years.
- Initially confirmed to have ICH, with time from onset to surgery ≤72 hours.
- Meeting the diagnostic criteria listed above and undergoing surgical treatment.
- Postoperative consciousness sufficiently clear to cooperate with cognitive and quality of life assessments.
- Informed consent obtained from the patient and their family.

### Exclusion criteria:

- History of cognitive impairment, psychiatric illness, or neurodegenerative disease.
- Presence of other serious neurological or systemic diseases, such as severe cardiac disease, hepatic or renal dysfunction.
- Severe hearing or visual impairments that would interfere with MSS.
- Withdrawal from the study or inability to complete the prescribed assessments and training protocols.

### Treatment methods:

Routine Postoperative Treatment and Nursing Care: Blood pressure and blood glucose levels were closely monitored and controlled within the normal range. For hypertensive patients, appropriate antihypertensive agents such as nifedipine or captopril were administered based on individual hemodynamic profiles. Diabetic patients were treated with dietary interventions combined with hypoglycemic agents (*e.g*., metformin, insulin) as indicated. Anti-infective Management: Antibiotic therapy was tailored according to the patient’s clinical condition and infection risk. For patients with confirmed pulmonary infections, antibiotic regimens were adjusted based on sputum culture and drug sensitivity test results.

### Nutritional Support:

Nutritional therapy was implemented based on the patient’s nutritional status and metabolic requirements. For patients capable of oral intake, a diet rich in protein, vitamins and dietary fiber was provided. For those unable to eat orally, enteral nutrition via nasogastric tube or percutaneous gastrostomy was administered.

On the basis of postoperative treatment and care, the control group received standard rehabilitation guidance, which included basic physical function training and ADL training. In addition to the same standard care, the observation group received CRT and MSS. CRT included memory, attention, executive function and language training:

### Memory Training:

Techniques such as story recall and image-based memory exercises were used. Sessions were conducted twice per week, each lasting 15 min for three months.

### Attention Training:

Tasks included number cancellation exercises and auditory digit span repetition. Training was delivered twice weekly, 15 minutes per session, for three months.

### Executive Function Training:

Patients performed task sequencing and problem-solving exercises, twice per week for 15 minutes over three months.

### Language Training:

In addition to standard language rehabilitation, training focused on verbal expression, reading and writing was added, delivered twice weekly for 15 minutes over three months. MSS comprised visual, auditory, tactile, olfactory and gustatory stimulation:

### Visual Stimulation:

Patients were shown brightly colored images or videos once daily, with each session lasting 15 minutes for three months.

### Auditory Stimulation:

Various types of music and natural sounds were played once daily for 15 minutes for three months.

### Tactile Stimulation:

Patients received massage therapy and participated in tactile games once daily for 15 minutes for three months.

### Olfactory Stimulation:

Exposure to different scents (*e.g*., aromatherapy oils, scent cards) was provided once daily for 10 minutes for three months.

### Gustatory Stimulation:

Different flavored foods or beverages were offered three times per week, with each session lasting 10 minutes for three months. The two groups of patients were followed up for six months, and the follow-up work of all patients was completed by the same group of surgeons.

### Outcome measures:

### Cognitive Function:

It was assessed in both groups at one month and three months postoperative using the Montreal Cognitive Assessment(MoCA). The MoCA evaluates multiple cognitive domains, including attention, memory, language, visuospatial abilities and executive function, with a total score of 30 points. Higher scores indicate better cognitive function. Additionally, the Mini-Mental State Examination(MMSE) was used to assess orientation, memory, attention, calculation ability, language skills and visuospatial function.

### Quality of life:

At three months postoperative, the 36 items Short Form Health Survey (SF-36)^9^ was used to evaluate patients’ quality of life. The SF-36 encompasses eight dimensions, including physical functioning, role limitations due to physical problems, bodily pain, general health, vitality, social functioning, role limitations due to emotional problems and mental health. Higher scores reflect better quality of life.

### Mental state:

It was assessed at one month and three months postoperative using the Self-Rating Anxiety Scale(SAS) and the Self-Rating Depression Scale(SDS). Each scale consists of 20 items rated on a 4-point Likert scale. The raw score is multiplied by 1.25 and rounded down to obtain the standardized score. For SAS, a standardized score of 50-59 indicates mild anxiety, 60-69 moderate anxiety and ≥70 severe anxiety. For SDS, a standardized score of 53-62 indicates mild depression, 63-72 moderate depression and ≥73 severe depression. All questionnaire were given to the participants for investigation, and then collected in about thirty minutes.

### National Institutes of Health Stroke Scale (NIHSS) Score:

Changes in NIHSS scores were compared between the two groups to evaluate the effectiveness of rehabilitation interventions in promoting neurological recovery.

### Statistical analysis:

Statistical analysis was performed using SPSS22.0. According to the data of each indicator in the pre-survey, the sample size is estimated by 95% confidence interval, and the largest one is the sample size of the study. The sample size required for each group was ≥40 cases on the basis of Fisher exact probability. Continuous variables were expressed as mean ± standard deviation (*x̅±s*) and between-group comparisons were conducted using independent sample t-tests. Categorical variables were expressed as frequencies (*n*) and percentages (%) and comparisons between groups were performed using the chi-square (χ²) test. A P-value of less than 0.05 was considered statistically significant.

## RESULTS

At one and three months after surgery, the MoCA scores of the observation group were significantly higher than those of the control group (*P<* 0.05, respectively). Similarly, at three months postoperative, the observation group also showed significantly higher scores on the SF-36 quality of life assessment compared with the control group (*P<* 0.05) (Table-I and II).

**Table-I:** Comparison of MoCA scores between groups at one and three months postoperative (*x̅*±*s*).

Group	MoCA at 1 month postoperative	MoCA at three months postoperative
Observation (n = 40)	22.5±3.2	25.8±3.1
Control (n = 40)	19.8±3.5	22.1±3.3
t-value	4.23	5.12
P-value	<0.05	<0.05

**Table-II:** Comparison of MMSE scores between groups at one and three months postoperative (*x̅*±*s*).

Group	MMSE score at 1 month postoperative	MMSE score at three months postoperative
Observation (n = 40)	23.1±2.8	26.4±2.7
Control (n = 40)	20.5±3.0	23.2±2.9
*t-value*	4.15	5.08
*P-value*	<0.05	<0.05

At three months postoperative, the observation group demonstrated significantly higher scores across all domains of the SF-36 compared with the control group (*P<* 0.05), indicating better quality of life (Table-III). At one and three months after surgery, the observation group had significantly lower SAS and SDS scores compared with the control group (*P <* 0.05, respectively) (Table-IV). At one and three months postoperative, the NIHSS scores of the observation group were significantly lower than those of the control group (*P <* 0.05, respectively) (Table-V).

**Table-III:** Comparison of SF-36 scores between groups at three months postoperative (*x̅*±*s*).

Group	Physical functioning	Role limitations due to physical problems	Social functioning	Role limitations due to emotional problems	Mental health	Bodily pain	Vitality	General health
Observation (n = 40)	78.5±10.2	75.3±9.8	80.1±11.0	76.2±10.5	79.4±10.3	77.1±10.7	75.8±10.4	78.2±10.6
Control (n = 40)	65.2±11.5	62.1±10.8	68.3±12.0	64.5±11.2	67.8±11.0	66.4±11.3	63.9±11.1	65.7±11.4
*t-value*	4.32	4.18	4.25	4.30	4.28	4.21	4.19	4.26
*P-value*	<0.05	<0.05	<0.05	<0.05	<0.05	<0.05	<0.05	<0.05

**Table-IV:** Comparison of SAS and SDS scores between groups at one and three months postoperative (*x̅*±*s*).

Group	SAS score at 1 month postoperative	SAS score at three months postoperative	SDS score at 1 month postoperative	SDS score at three months postoperative
Observation (n = 40)	42.3±6.5	38.1±6.2	43.2±6.8	39.5±6.4
Control (n = 40)	50.1±7.2	46.8±7.0	51.3±7.5	47.9±7.3
*t-value*	4.10	4.05	4.08	4.02
*P-value*	<0.05	<0.05	<0.05	<0.05

**Table-V:** Comparison of NIHSS scores between groups at one and three months postoperative (n[%]).

Group	NIHSS score at 1 month postoperative (*x̅*±*s*)	NIHSS score at three months postoperative (*x̅*±*s*)
Observation (n = 40)	13.20±1.20	10.50±1.10
Control (n = 40)	15.80±1.30	13.40±1.20
*t-value*	10.25	12.10
*P-value*	<0.001	<0.001

## DISCUSSION

This study focused on the effect of CRT plus MSS on patients after ICH surgery. The results demonstrated that this combined intervention significantly improved cognitive function, quality of life and mental state, while also reducing NIHSS scores. In terms of cognitive function, the observation group showed significantly higher MoCA scores than the control group at both one and three months postoperative. CRT specifically targets the enhancement of neural pathways related to cognition, while MSS promotes neural plasticity through multi-channel sensory input. The synergy between these two approaches facilitates more effective cognitive recovery.^10^,^11^ For instance, attention training involving number cancellation and auditory digit span repetition integrates visual and auditory stimuli, thereby engaging multiple brain regions simultaneously and improving attentional focus and maintenance. From a neurological perspective, MSS activates various sensory processing areas, such as the visual and auditory cortices, which are extensively connected to brain regions responsible for cognitive function. This broad neural activation supports cognitive enhancement.^12^ Research has shown that neural plasticity persists into adulthood and can be particularly responsive following brain injury. Targeted interventions have the potential to induce neural remodeling.^13^ The combined intervention model used in this study leverages this neuroplastic potential, creating favorable conditions for cognitive rehabilitation after ICH surgery.

In terms of quality of life, at three months postoperative, the observation group scored significantly higher than the control group across all dimensions of the SF-36. Improved cognitive function enabled patients to perform ADL more independently, such as dressing, eating and personal hygiene, which directly contributed to better scores in the physical functioning domain.^14^ Furthermore, enhanced psychological well-being encouraged greater participation in social interactions, thereby improving social functioning scores.^15^ Previous studies have demonstrated a strong association between cognitive function and quality of life, with cognitive impairment limiting social engagement and reducing life satisfaction.^16^ In the present study, the combined intervention effectively broke this barrier by improving cognitive performance, thereby enhancing patients’ overall quality of life.

Regarding mental state, the observation group exhibited lower SAS and SDS scores than the control group at one and three months postoperative. Patients with physical impairments resulting from ICH are prone to developing anxiety and depression.^17^ With the CRT + MSS approach, patients could perceive progressive cognitive improvement, thereby enhancing their confidence in recovery and alleviating psychological stress. Additionally, the pleasant experiences associated with MSS (*e.g*., listening to favorite music, viewing aesthetically pleasing images) may modulate neurotransmitter secretion in the brain. For example, such stimuli can increase dopamine release, thereby improving mood and emotional regulation.^18^ Related studies suggest that positive emotional states can enhance psychological resilience, enabling patients to better cope with the mental challenges posed by illness.^19^

With respect to neurological function, the NIHSS scores of patients in the observation group were significantly lower than those in the control group at both one and three months postoperative (*P <* 0.05, respectively), indicating that the combined approach effectively reduced neurological impairment. This may be attributed to the ability of MSS to activate multiple sensory pathways in the brain, thereby enhancing neural plasticity and promoting functional reorganization.^20^ Concurrently, CRT reinforced cognitive recovery and the synergistic effect of the two interventions provided strong support for neurological recovery following ICH surgery. Moreover, the fact that significant improvements were observed as early as one month postoperative suggests that this combined intervention modality exhibits considerable value in the early phase of rehabilitation.^21^

### Limitations:

Despite the positive outcomes of this study, several limitations should be acknowledged. First, the modest sample size may affect the generalizability and reliability of the findings. Future studies should aim to include larger and more diverse populations, covering different age groups, hemorrhage locations and disease severities, to enhance statistical power and the robustness of conclusions. Second, the follow-up period in this study was relatively short, with observations limited to three months postoperative. Longer-term follow-up is needed to determine the sustained effects of the intervention on cognitive function and quality of life, thereby evaluating its long-term efficacy. Third, this study did not explore the underlying neural mechanisms of the combined intervention in depth.

## CONCLUSIONS

CRT combined with MSS represents an effective intervention for postoperative patients with ICH. This combined approach can significantly improve cognitive function, quality of life and psychological well-being while reducing neurological impairment. Despite some limitations, the findings of this study offer valuable insights into postoperative rehabilitation strategies for patients with ICH. Through the synergistic effects of MSS and CRT, this combined approach can facilitate neural recovery and reorganization, providing a strong theoretical and practical foundation for clinical rehabilitation.

### Authors’ Contributions:

**YL:** Literature search, Conceived and designed the study.

**XL:** Collected the data and performed the analysis. Critical Review.

**BZ:** Writing of the manuscript and is responsible for the integrity of the study.

All authors have read and approved the final manuscript.
